# Long-Term Sodium Ferulate Supplementation Scavenges Oxygen Radicals and Reverses Liver Damage Induced by Iron Overloading

**DOI:** 10.3390/molecules21091219

**Published:** 2016-09-16

**Authors:** Yang Qiao, Huan He, Zeyu Zhang, Zhangping Liao, Dong Yin, Dan Liu, Bo Yi, Ming He

**Affiliations:** 1 Jiangxi Provincial Institute of Hypertension, The First Affiliated Hospital of Nanchang University, Nanchang 330006, China; YangQ123@163.com; 2 Jiangxi Provincial Key Laboratory of Basic Pharmacology, Nanchang University School of Pharmaceutical Science, Nanchang 330006, China; hhmm56@bu.edu (H.H.); jxnczzy777@163.com (Z.Z.); liaozp1980@163.com (Z.L.); 3 Jiangxi Provincial Key Laboratory of Molecular Medicine at the Second Affiliated Hospital, Nanchang University, Nanchang 330006, China; dongyin24@126.com; 4 Second Abdominal Surgery Department, Jiangxi Province Tumor Hospital, Nanchang 330029, China

**Keywords:** iron overload, taurine, sodium ferulate, liver damage, oxidative stress

## Abstract

Ferulic acid is a polyphenolic compound contained in various types of fruits and wheat bran. As a salt of the active ingredient, sodium ferulate (SF) has potent free radical scavenging activity and can effectively scavenge ROS. In this study, we examined the effect of SF on iron-overloaded mice in comparison to a standard antioxidant, taurine (TAU). We determined the protective role of SF against liver injury by examining liver-to-body ratio (%), transaminase and hepatocyte apoptosis in rats supplied with 10% dextrose intraperitoneal injection. In addition, antioxidative enzymes activities, ROS formation, mitochondrial swelling, and mitochondrial membrane potential (MMP) were all evaluated to clarify the mechanism of protective effect of SF associated with oxidative stress. After 15 weeks of SF treatment, we found a significant reduction in liver-to-body weight radio and elevation in both transaminase and hepatocyte apoptosis associated with iron-injected to levels comparable to those achieved with TAU. Both SF and TAU significantly attenuated the impaired liver function associated with iron-overloaded in mice, whereas neither showed any significant effect on the iron uptake. Furthermore, treatment with either SF or TAU in iron-overloaded mice attenuated oxidative stress, associated with elevated oxidant enzymes activities, decreased ROS production, prevented mitochondrial swelling and dissipation of MMP and then inhibited hepatic apoptosis. Taken together, the current study shows that, SF alleviated oxidative stress and liver damage associated with iron-overload conditions compared to the standard ROS scavenger (TAU), and potentially could encourage higher consumption and utilization as healthy and sustainable ingredients by the food and drink.

## 1. Introduction

Hepatic iron overload resulting from hemochromatosis or hematologic disorders such as thallasemia can cause cirrhosis, end-stage liver disease and hepatocellular carcinoma [1,2]. A common feature of both acute and chronic liver diseases is the excessive death of hepatocytes, leading to the loss of liver function and eventually liver failure [3,4]. Considerable attention has focused on the hypothesis that iron-catalyzed oxidative stress plays a major role in hepatocyte death and liver injury [5].

Taurine (TAU) is a sulphur-containing amino acid that stabilises cell membrane and possesses several physiological roles [6,7,8]. There are many reports on TAU’s protective effects against different chemically-induced hepatotoxicity [9,10]. Furthermore, it has been reported that this amino acid could scavenge free radicals and act as an antioxidant in iron-overloaded liver [11]. Hence, the protective effects of TAU could be due to the antioxidant capability of this amino acid.

Likewise, ferulic acid, because of its phenolic nucleus and unsaturated side chain, can readily form a resonance stabilized phenoxy radical, has potent free radical scavenging activity and can effectively scavenge ROS [12,13]. Ferulic acid is one of the ubiquitous compounds in diet, especially rich in grains, fruits, vegetables and beverage [14,15]. Sodium ferulate (SF) is the sodium salt of ferulic acid. Ferulic acid is unstable and can’t be easily dissolved in water. Compared with ferulic acid, SF is more stable and more easily dissolved in water. It has been shown that SF has a broad spectrum of biological activities, such as anti-inflammatory, antioxidative, and antimutagenic effects [16,17,18,19], so in the present study SF was used to treat iron-overloaded mice.

Although SF and ferulic acid exhibit beneficial effects in many diseases, whether SF or ferulic acid has beneficial effects on liver damage in iron-overloaded mice is still unclear. In the present study, the potential protective role of SF on liver damage associated with iron-overloaded conditions compared to a standard antioxidant, TAU and also to identify the possible mechanisms underlying this effect. This will identify a new preventive strategy for limiting liver damage under iron-overloaded conditions.

## 2. Results

### 2.1. SF Attenuates Mice Liver Damage Induced by Iron Overload

Administration of iron to mice over a 4-months time course successfully increased iron concentration of serum and liver, and produced marked liver damage specified as the significant increases of LW/BW and serum ALT and AST levels to that found in the control group. Oral administration of TAU and SF (40 and 30 mg/kg/day, respectively) significantly attenuated the developed liver damage, resulting in decreases of LW/BW and serum ALT and AST levels (both at *p* < 0.01). However, neither TAU nor SF significantly affected iron concentration of serum and liver (Table 1).

### 2.2. SF Reduces Hepatocyte Apoptosis

**Figure 1:**
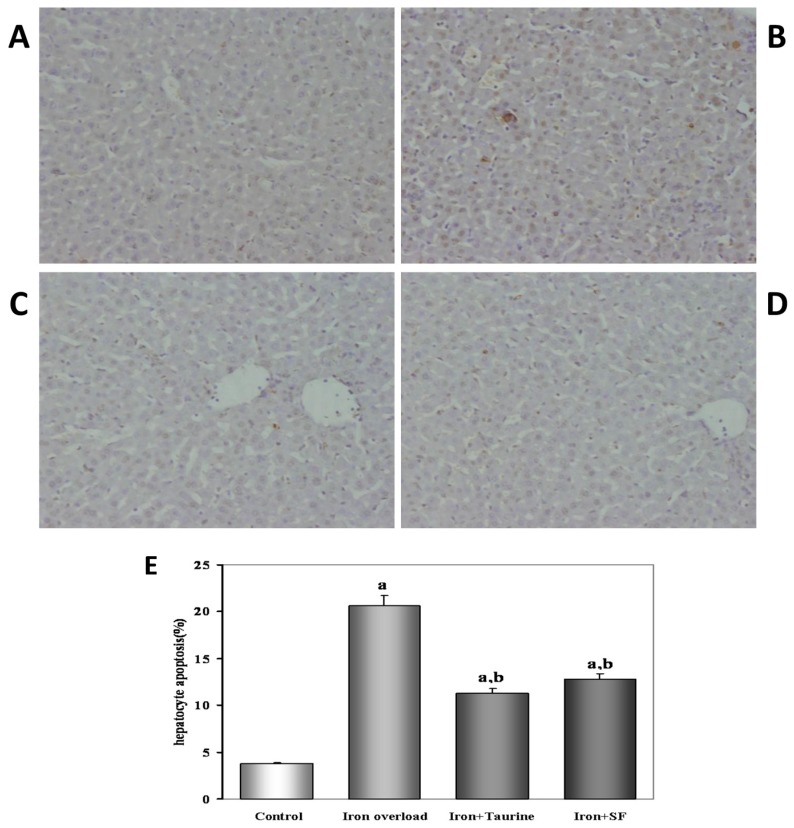
The effect of TAU or SF on iron overload-induced hepatocyte apoptosis. TUNEL-positive cells are identified as apoptotic, and arrowheads indicate the TUNEL-positive hepatocytes in representative photomicrographs. Original magnification is ×400. (**A**) control group; (**B**) iron overload group; (**C**) TAU-treated group; (**D**) SF-treated group (**E**) Quantitative analysis of apoptotic hepatocytes expressed as percentage of TUNEL-positive nuclei in hepatocytes. Values are mean ± SE (*n* = 12). a: *p* < 0.01, vs. control group; b: *p* < 0.01, vs. iron overload group.

Ferrocene supplement in the diet significantly promoted hepatocyte apoptosis when compared to the control group, as indicated by a significant increase in the TUNEL-positive hepatocytes. Surprisingly, both TAU and SF treatment showed a significant decrease in the TUNEL-positive hepatocytes when compared to the iron-overload group (both at *p* < 0.01) (Figure 1). Thus, both TAU and SF prevent hepatocyte apoptosis associated with iron-overload conditions.

### 2.3. SF Elevates Enzymatic Antioxidants Activity

**Figure 2:**
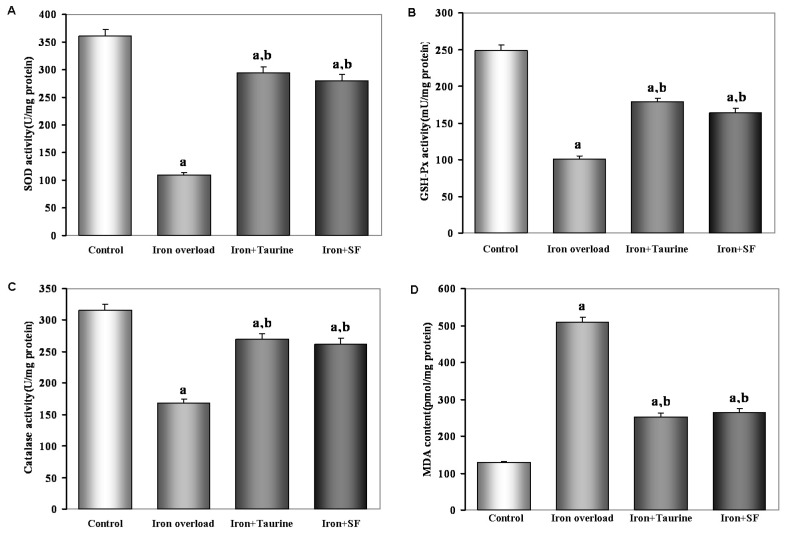
The effect of TAU or SF on the activities of the antioxidant enzymes and lipid peroxidation in iron-overloaded mice. (**A**) SOD; (**B**) GSH-Px; (**C**) catalase; (**D**) MDA. Data represents mean ± SE of 12 independent experiments. a: *p <* 0.01, vs. control group; b: *p <* 0.01, vs. iron overload group.

As seen from Figure 2, the activities of SOD, GSH-Px and catalase are lower, but MDA level is higher in the iron-overloaded hepatic tissues relative to no-treated hepatic tissues (*p* < 0.01). However, both TAU and SF exert same effects on the oxidant and antioxidant parameters in the iron-overloaded hepatic tissues. They caused significant increases in SOD, GSH-Px and catalase activities and decreases in MDA levels. There were no significant differences in these parameters between the two groups.

### 2.4. SF Increases Non-Enzymatic Antioxidants

**Figure 3:**
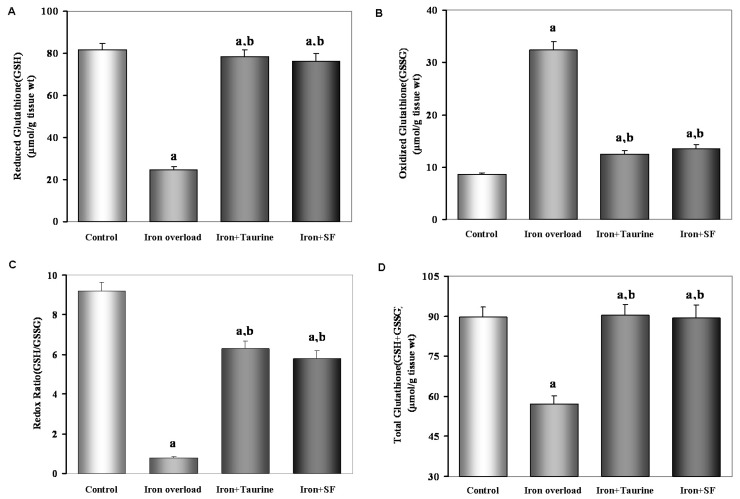
The effect of TAU or SF on hepatic GSH (**A**) and GSSG (**B**) levels, the ratio of GSH/GSSG (**C**) and the total amount GSH + GSSG (**D**) in iron-overloaded mice. Data represents mean ± SE of 12 independent experiments. a: *p <* 0.01, vs. control group; b: *p <* 0.01, vs. iron overload group.

Exposure to iron-overload condition significantly decreased GSH contents (Figure 3A), the ratio of GSH/GSSG (Figure 3B) and the total amount (GSH + GSSG) (Figure 3D) compared with control group (*p* < 0.01). Surprisingly, both TAU and SF exhibited a significant increase in GSH contents (Figure 3A), the ratio of GSH/GSSG (Figure 3C), and the total of GSH + GSSG (Figure 3D) when compared to the iron-overload group. This suggested TAU and SF could reverse oxidative stress induced by excessive iron by elevating non-enzymatic antioxidants.

### 2.5. SF Reduces the ROS Production

**Figure 4:**
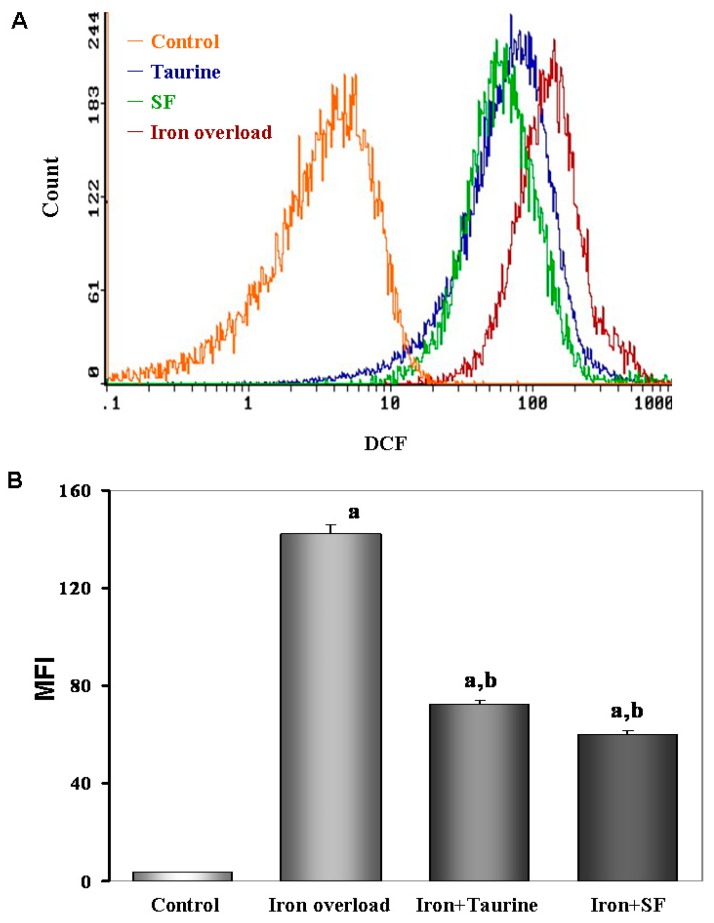
The effect of TAU or SF on hepatic intercellular ROS generation in iron-overloaded mice. (**A**) Cellular ROS contents were measured by flow cytometry. The incubation methods of every group were the same as we described in the Materials and Methods. Hepatocytes were then cultured for 20 min in the dark. All cells harvested for DCFH-DA incubating and measured using flow cytometry system; (**B**) Quantitative analysis of mean fluorescence intensity (MFI) of DCF. Data represents mean ± SE of 12 independent experiments. a: *p <* 0.01, vs. control group; b: *p <* 0.01, vs. iron overload group.

We next investigated if oral administration of SF can inhibit the production of ROS induced by iron overload in mice. As shown in Figure 4, ferrocene feeding for 4 months resulted in a ROS curve shift to the right, which means iron-overload increased the level of ROS (*p* < 0.01) compared to the control group. However, oral administration of TAU and SF significantly reduced the elevated ROS levels (both at *p* < 0.01), and caused the curve of ROS to shift to the left.

### 2.6. SF Inhibits the Mitochondrial Swelling

**Figure 5:**
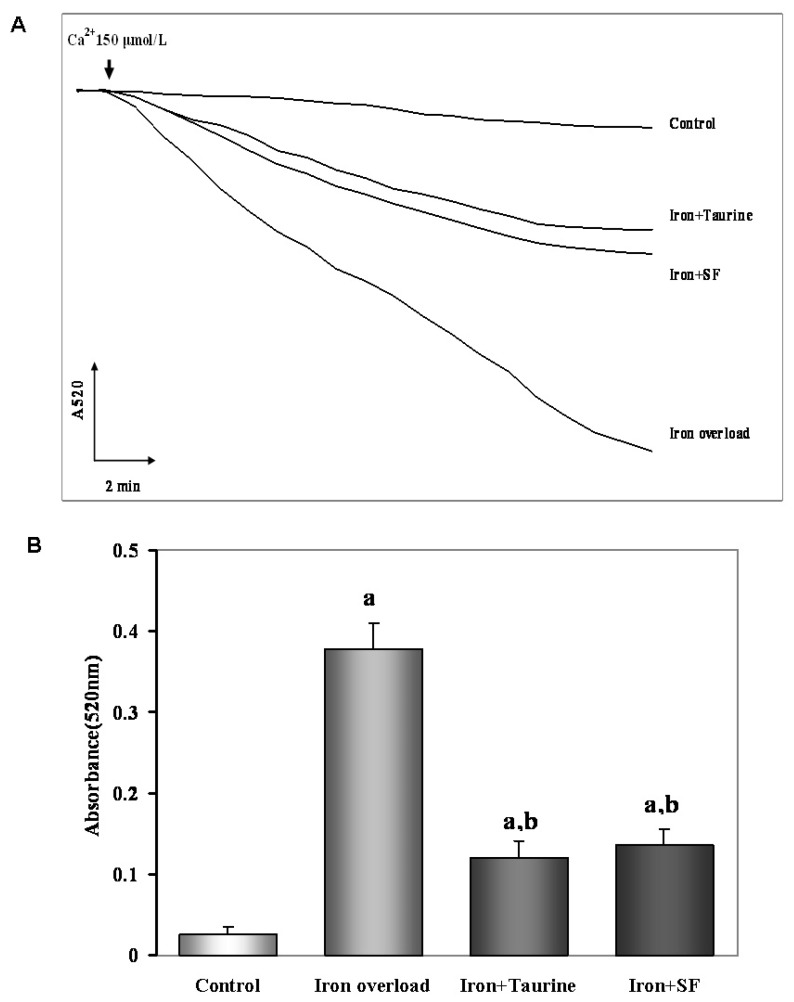
The effect of TAU or SF on hepatocyte mitochondrial swelling in iron-overloaded mic Data represents mean ± SE of 12 independent experiments. (**A**) Ca^2+^-induced mitochondrial swelling in four groups with different treatment. During the onset of mitochondrial swelling, 150 μM Ca^2+^ was added to initiate the reaction. Iron-overload induced mitochondrial swelling, resulted in the large decrease in the absorbance of the mitochondrial suspension at 520 nm in 2 min. However, both SF and TAU inhibited the swelling process; (**B**) Quantitative analysis of mitochondrial swelling. a: *p <* 0.01, vs. control group; b: *p <* 0.01, vs. iron overload group.

To elucidate the molecular mechanisms by which TAU and SF maintained hepatocyte MMP and declined oxidative stress, we measured the hepatocyte mitochondrial swelling using the method as mentioned before. Mitochondrial swelling was obtained by the addition of 150 µM Ca^2+^. The results showed that iron-overload promote mitochondrial swelling, whereas, TAU and SF treatment both completely inhibited Ca^2+^ induced mitochondrial swelling in isolated mouse liver mitochondria (both at *p* < 0.01). This result indicated that the mechanism of protective action against MMP collapse could be considered for SF which inhibit mitochondrial swelling (Figure 5).

### 2.7. SF Inhibits the Loss of MMP (Mitochondrial Membrane Potential)

**Figure 6:**
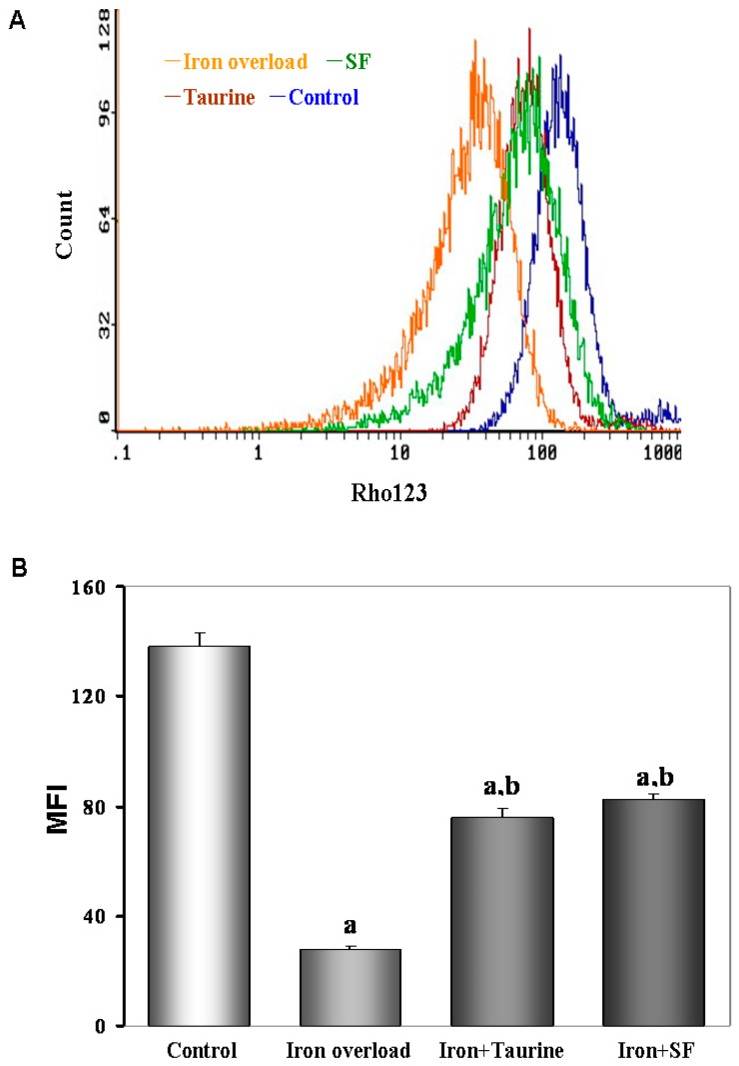
The effects of TAU or SF on mitochondrial membrane potential (MMP) in iron-overloaded mice. (**A**) Fluorescent indicator Rhodamine123 was used to detect the change of MMP in different gourps. The fluorescent distribution curve shifted to the left in iron overload group, indicating a reduction of MMP. However, the curve shifted to the right in the cells treated with ST or TAU; (**B**) Quantitative analysis of mean fluorescence intensity (MFI) of Rhodamine123. Data represents mean ± SE of 12 independent experiments. a: *p <* 0.01, vs. control group; b: *p <* 0.01, vs. iron overload group.

Rhodamine 123 (Rh123) is a lipophilic compound that accumulates inside mitochondria, and can be used to analyze, at least qualitatively, the value of mitochondrial membrane potential. Then the hepatocytes were incubated with Rh123 according to the previously described method. As show in Figure 6, iron overload shifted the curve of MMP to the left and induced MMP depolarization compared to the control group, whereas TAU and SF prevented MMP dissipation and shifted the curve back to the right.

## 3. Discussion

This study presented, for the first time, the potential protective of SF against iron-overload condition, the main active components of the ferulic acid compared to the standard antioxidant, TAU. This is supported by the following findings: (1) SF reversed iron overload-induced hepatic damage without reducing iron deposition, (2) Enhanced oxidant stress following iron overload in liver was ameliorated by SF and (3) SF reduced mitochondrial swelling and membrane potential associated with oxidant stress. Thus, it is suggested that the overall protective role of SF against iron overload is mediated by inhibiting oxidant stress and maintaining mitochondrial membrane potential.

In our study, we considered the most reliable model of iron overload in mice by including ferrocene (0.2% for 90 days and then 0.4% for the remaining 30 days) in diet. This was sufficient to significantly develop iron-overload in 120 days. Also, the iron-overloaded animal group was characterized by increasing in LW/BW, serum ALT and AST, TUNEL-positive hepatocytes, which is consistent with previous studies [20]. Elevated LW/BW and serum ALT and AST might be correlated to impaired liver function. Whereas, increased TUNEL-positive hepatocytes is referred to the aggravated hepatocyte apoptosis in the affected animals. Our results indicated that both SF and TAU significantly reduced the aggravations in liver damage associated with iron overload. The effect of SF is explained by its antioxidation, which is consistent with previous studies that reported that antioxidant protects against liver damage in iron-overloaded mice models [20,21,22].

Iron-catalyzed oxidative damage to the liver is presumed to be the major causal factor in the impairment of hepatic function [23,24]. Therefore, we speculated that the mechanisms by which TAU and SF prevent iron overload-induced liver damage can be attributed to inhibition of oxidant stress. In the present study, both TAU and SF prevented the excessive ROS generation in iron-overloaded mice. Antioxidant compounds are capable of neutralizing ROS and counteract the harmful effects of ROS [25,26,27]. In this study, antioxidant measurements were performed in all four groups. Statistically significant differences were found for all antioxidant measurements (SOD, GSH-Px, Catalase activities and GSH, GSSH levels in hepatic tissue, and MDA level in hepatic tissue) between the four groups. In the iron-overload group, SOD, GSH-Px, Catalase and GSH values were lower than in the control group. Moreover, except for MDA, antioxidant measurement values were higher than in the iron-overload group. MDA was a typical biomarker used in the evaluation of injury due to lipid peroxidation, which represented the most frequent injury resulting from the activation of ROS [28,29]. Therefore, TAU and SF significant prevented the MDA increase associated with iron overload.

In view of the importance of mitochondria in control of cell fate [30,31], we also highlighted the direct effects of SF on isolated mouse liver mitochondria. In iron-overload animal group, we observed an aggravated mitochondrial swelling and dissipated MMP. On the other hand, animals treated with TAU and SF prevented the aggravation of mitochondrial swelling and elimination of MMP, which are consistent with inhibiting excessive ROS generation and enhancing antioxidant activities. These findings suggested that SF might prevent the iron overload-induced liver damage by suppressing oxidative stress and stabilizing MMP, as the standard antioxidant TAU.

In conclusion, SF, with its antioxidant properties, could increase antioxidant enzymes and lead to a decrease in oxidative stress associated with iron overload. Because SF is an herbal product, and the adverse reactions were limited, so it might be used as a nutritional supplement in people with iron-overload condition, such as hemochromatosis or hematologic disorders. These products could be considered as products that will enhance the quality of life.

## 4. Materials and Methods

### 4.1. Animals

Forty-eight male Kunming mice weighing 15.1 g ± 0.5 g from Nanchang University (Nanchang, China) were maintained in clear polypropylene cages in stable environmental conditions and equal light–dark cycles. Mice had free access to a commercially available rodent-pellet diet and purified water. Animal care in this study conformed to the National Institutes of Health (NIH) Guide for Care and Use of Laboratory Animals (NIH publication 86–23, revised 1986). Experimental protocol was approved by the Nanchang University Ethical Committee for Animal Handling.

### 4.2. Study Protocol

Animals were randomly divided into four experimental groups (12 mice/group) as follows: control, iron-overload, TAU-treated and SF-treated. Iron-overload mice were fed for 4 months on a pellet diet (AIN-93G) supplemented with iron in the form of ferrocene. The proportion of iron in the diet was maintained at 0.2% (*w*/*w*) for 90 days and then to 0.4% (*w*/*w*) for the remaining 30 days [32]. Then, TAU and SF (20 and 15 mg/kg/day, respectively) were administered daily by oral gavage prior to iron-overload administration 6 weeks and throughout the course of the experiments. Control mice were fed the AIN-93G diet without iron, TAU and SF. After experiment, mice were euthanized by cervical dislocation, and blood was collected by cardiac puncture. The liver was immediately excised, weighed and divided for analysis as following methods.

### 4.3. Measurement of Biochemical Parameters and Indices of Liver Injury

Serum iron concentrations were determined using the assay based on the generation of an iron-ferrozine complex, as described previously [33]. Iron concentrations were expressed as micrograms of iron per gram of dry weight of liver and measured spectrophotometrically at 535 nm, following reaction with 2 mM bathophenanthroline disulfonic acid [34]. LW/BW (liver weight/body weight) was calculated according to the formula: LW/BW = 100 × (liver weight (g)/Body weight (g)). Serum AST and ALT activities were measured using an autoanalyser (Cobas Integra 400, Roche, Holliston, MA, USA) and an ALT/AST reagent kit from Roche Diagnostics (Indianapolis, IN, USA).

### 4.4. Determination of Antioxidant Enzymes Activities and Lipid Peroxidation

The protein content of the liver was measured according to the methods of Lowry et al. [35] using bovine serum albumin as a standard. Malondialdehyde (MDA, pmol/mg) level, CAT (U/mg), SOD (U/mg), and GSH-Px (mU/mg) enzyme activities were measured, respectively [36,37,38]. MDA levels were measured by the thiobarbituric acid reactive substances method [39]. SOD activity was measured on the basis of the inhibition of nitroblue tetrazolium (NBT) reduction by O_2_ generated by the xanthine/xanthine oxidase system [36]. One unit for SOD activity was expressed as the enzyme protein amount causing 50% inhibition in the NBT reduction rate. CAT activity was determined by measuring the absorbance decrease of H_2_O_2_ at 240 nm [37]. GSH-Px activity was measured by following changes in nicotinamide adenine dinucleotide phosphate (NADPH) absorbance at 340 nm [38].

### 4.5. Estimation of Non-Enzymatic Antioxidants (GSH and GSSH)

To evaluate if SF can maintain hepatic GSH homeostasis and elevate GSH level, hepatic concentrations of reduced GSH and GSSG were measured by GSH and GSSG assay kit purchased from Beyotime Institute of Biotechnology (Haimen, Jiangsu, China). Briefly, after treatment, 0.1 g of liver was homogenized with a pellet pestles-Cordless motor (Sigma-Aldrich, St. Louis, MO, USA) and the supernatants were collected by centrifugation at 10,000× *g* for 10 min at 4 °C, and the concentrations of GSH and GSSG were measured according to the instructions of the assay kit. All biochemical determinations were normalized to the protein content using bicinchoninic acid method (Pierce, Rockford, IL, USA).

### 4.6. Hepatocyte Preparation

Hepatocytes were isolated using a two-step collagenase perfusion method as previously described. Following mechanical disruption of the liver capsule, liver cells were collected in Williams’ Medium E and serially filtered (30-, 50- and 80-mesh) through an 85-mL Cellector (Bellco Biotechnology, Vineland, NJ, USA) tissue sieve. Between 10 × 106 and 25 × 106 cells were obtained from a single mouse liver.

### 4.7. Measurement of Intracellular ROS

A fluorescent probe (Beyotime, Shanghai, China) was used to detect intracellular ROS level. Hepatocytes were washed twice with cold phosphate-buffered saline (PBS) and incubated in Dulbecco's Modified Eagle’s Medium containing 10 mmol/L 2′,7′-dichlorofluorescein diacetate (DCFH-DA) for 20 min at 37 °C. DCFH-DA is cell permeable and can be rapidly oxidized into highly fluorescent 2′,7′-dichlorofluorescein (DCF) by ROS. The fluorescence intensity of each group was determined using flow cytometric analysis (Becton-Dickinson, Franklin Lakes, NJ, USA) at excitation and emission (ex/em) wavelengths of 485/528 nm, respectively.

### 4.8. Determination of Mitochondrial Membrane Potential (MMP)

Loss of the mitochondrial membrane potential (MMP) was assessed with flow cytometry using the fluorescent indicator Rhodamine123 (Rho123; Sigma-Aldrich, St. Louis, MO, USA). In Brief, the hepatocytes were transferred to 1.5 mL Eppendorf tubes and kept no ice. After washed twice with ice-cold PBS, the pellet was gently resuspended and adjusted to a concentration of 1 × 10^6^ cells/mL. The suspensions were incubated with rhodomine123 (10 M) for 30 min in the darkness at 4 °C. Then the cells were washed twice with ice-cold PBS and kept on ice in the dark until analyzed with flow cytometer at 37 °C, and were immediately submitted for flow analysis. At least 10,000 events were acquired per sample.

### 4.9. Isolation of Mitochondria from Mouse Liver

Mitochondria were isolated from mouse liver by differential centrifugation [40]. In brief, mouse liver was homogenized using a Potter-Elvehjem glass homogenizer (Tiandz, Beijing, China). Homogenates were then centrifuged for 5 min at 600× *g* and the resulting supernatant further centrifuged for 10 min at 11,000× *g*. The pellet was resuspended and the same centrifugation process was repeated. Pellets were then suspended and centrifuged for 15 min at 3400× *g*. The final mitochondrial pellet was suspended in storage buffer consisting of 250 mM sucrose and 10 mM HEPES-KOH (4 °C, pH 7.4) and used within 3 h.

### 4.10. Evaluation of Mitochondrial Swelling

The swelling experiments were performed as a previously described methed [41] using a standard medium containing 125 mM sucrose, 10 mM HEPES buffer (pH 7.2), 2.5 mM succinate and 4.0 mM rotenone at 25 °C. The final volume used was 1.0 mL, and the protein concentration was ~0.5 mg/mL. Then, swelling was initiated by adding 150 μM CaCl_2_. Swelling was estimated from the decrease in absorbance at 520 nm with a spectrometer (Hitachi U 2000, Hitachi, Tokyo, Japan).

### 4.11. TUNEL Assay

The terminal deoxynucleotidyl transferase mediated nick-end labeling (TUNEL) assay was performed to detect hepatocyte apoptosis. The hepatocytes were plated on glass Lab-Tek Chamber slides (Sigma) and washed with PBS, then fixed in 1% paraformaldehyde for 10 min. These were then postfixed in pre-cooled ethanol–acetic acid (2:1) for a further 5 min at –20 °C. Following washing with PBS, the cells were incubated with a TUNEL reaction buffer at 37 °C for 1 h in a humidified chamber. As a positive control, cells were treated with DNase I (1.0 mg/mL, Sigma) for 10 min, to introduce nicks into the genomic DNA. Brown colored cells represent apoptotic cells in each glass-slide under the high magnification (×400) microscope (OLYMPUS, Tokyo, Japan). The percentage of hepatocyte apoptosis with DNA nick-end labeling was determined by counting the number of cells exhibiting brown nuclei among 1000 cells in duplicate plates.

### 4.12. Statistical Analysis

All statistical analysis was performed with Statistical Product and Service Solutions 13.0 software (SPSS 13.0, SPSS, Chicago, IL, USA). Data was represented as means ± SE. Statistical analysis was performed by Student-Newman-Keuls post-test for comparison between two groups, and one-way ANOVA for multiple groups. *p* < 0.05 was considered as statistically significant.

## Figures and Tables

**Table 1:** Effect of TAU or SF on the serum and hepatic iron concentration, liver-to-body weight ratio, serum levels of ALT and AST in iron-overloaded mice.

Group	Control	Iron	Iron + TAU	Iron + SF
Serum iron concentration (μmol/L)	35.03 ± 1.26	415.26 ± 14.21 ^a^	395.18 ± 15.18 ^a^	398.64 ± 16.21 ^a^
Hepatic iron concentration (mg/g dry weight)	0.058 ± 0.002	1.102 ± 0.032 ^a^	1.081 ± 0.029 ^a^	1.076 ± 0.031 ^a^
LW/BW (mg/g)	45.6 ± 2.2	102.6 ± 4.3 ^a^	62.3 ± 2.2 ^a,b^	65.1 ± 2.6 ^a,b^
ALT (U/L)	46.82 ± 1.35	235.21 ± 8.62 ^a^	120.32 ± 4.61 ^a,b^	135.06 ± 5.25 ^a,b^
AST (U/L)	100.65 ± 3.25	405.28 ± 13.06 ^a^	224.52 ± 9.31 ^a,b^	240.61 ± 9.53 ^a,b^

Note: Data are expressed as the mean ± the standard error of the mean (*n* = 12). ^a^ *p* < 0.01 vs. Control group; ^b^ *p <* 0.01 vs. Iron group. ALT, alanine transaminase; AST, aspartate transaminase.
